# *Lactiplantibacillus plantarum* HY7715 Attenuates Oxidative Stress-Induced Neurobiological Aging-Related Changes by Modulating Senescence-Associated Markers and Gut Microbiota

**DOI:** 10.4014/jmb.2604.04063

**Published:** 2026-06-02

**Authors:** Daehyeop Lee, Haeryn Jeong, Hyeonjun Gwon, Kippeum Lee, Joo-Yun Kim, Jae-Jung Shim, Jae-Hwan Lee

**Affiliations:** R&BD Center, hy Co., Ltd., 22, Gyeonggi-do 17086, Republic of Korea

**Keywords:** *Lactiplantibacillus plantarum* HY7715, Neurobiological aging, Cellular senescence, Neuroprotective, Gut-brain axis

## Abstract

External stressors can accelerate biological aging-related processes by promoting oxidative stress and senescence-associated molecular alterations in the brain. This study investigated the potential of *Lactiplantibacillus plantarum* HY7715 to attenuate oxidative stress-induced neurobiological aging-related changes using H_2_O_2_-induced HT22 hippocampal cells and a restraint-stressed mouse model. In HT22 cells, HY7715 reduced reactive oxygen species accumulation, decreased 8-hydroxy-2′-deoxyguanosine (8-OHdG) levels, and lowered the proportion of senescence-associated β-galactosidase-positive cells. These effects were accompanied by suppression of p53/p21 signaling and restoration of *Tert* expression. In restraint-stressed mice, HY7715 reduced the number of p21-positive cells in the hippocampus, significantly lowered *p53* expression, restored *Tert* expression, reduced *Il-6* expression, and improved antioxidant-related gene expression, including *Gpx1* and *Sod1*. Microbiome analysis showed that HY7715 reshaped the stress-altered gut microbiota toward a *Lactobacillus*-enriched profile and reduced the abundance of *Lachnospiraceae*, *Acetatifactor*, *Desulfovibrio*, and *Oscillibacter*. Collectively, these findings suggest that HY7715 may attenuate oxidative stress-induced neurobiological aging-related changes by modulating senescence-associated molecular markers and stress-altered gut microbiota, highlighting its potential as a candidate for supporting healthy brain aging.

## Introduction

With the global rise in life expectancy, the socioeconomic burden of neurodegenerative disorders continues to escalate, as aging remains their predominant risk factor [1, 2]. Beyond chronological aging, accumulating evidence indicates that external stress can promote biological aging-related processes within the central nervous system (CNS), thereby increasing neural vulnerability and stress-associated functional decline [3]. Among various experimental paradigms, restraint stress is widely used to induce prolonged psychosocial stress, leading to sustained activation of the hypothalamic-pituitary-adrenal (HPA) axis [4]. Chronic activation of this axis results in excessive glucocorticoid secretion, disrupting cellular and metabolic homeostasis and promoting oxidative imbalance [5]. This dysregulation contributes to mitochondrial dysfunction, genomic instability, and ultimately stress-induced premature senescence (SIPS) [6]. Senescent neural cells remain metabolically active but acquire a senescence-associated secretory phenotype (SASP), fostering a chronic pro-inflammatory microenvironment that contributes to cognitive decline and progressive neurodegeneration [7].

Mechanistically, stress-induced redox imbalance leads to excessive accumulation of reactive oxygen species (ROS) in brain tissue, including superoxide anion (O_2_^·-^), hydroxyl radical (·OH), and hydrogen peroxide (H_2_O_2_) [8-10]. These elevated ROS levels act as central mediators of SIPS by initiating and amplifying the DNA damage response (DDR) [11]. ROS-induced genomic damage activates key signaling pathways, including p53, which can further enhance mitochondrial ROS production, thereby establishing a positive feedback loop [12]. Concurrently, oxidative stress accelerates telomere shortening and induces telomere dysfunction, further amplifying DDR signaling and activating the p53/p21 pathway. This cascade enforces stable cell-cycle arrest and promotes the development of SASP, thereby contributing to the establishment and persistence of neurobiological senescence [13-15].

Given that oxidative stress and genomic instability are hallmarks of aging, therapeutic strategies aimed at restoring redox homeostasis have gained increasing attention. In this context, modulation of the gut microbiota has emerged as a promising approach for attenuating stress-related neurobiological aging [16]. We previously demonstrated that *Lactiplantibacillus plantarum* HY7715 alleviates stress-induced anxiety-like behaviors and restores mitochondrial function [17]. Nevertheless, its potential role in regulating DNA damage responses, telomere-associated regulation, and senescence-associated signaling under chronic stress conditions has not been fully elucidated. In this study, we investigated the potential of HY7715 to attenuate stress-induced neurobiological aging-related alterations by examining oxidative DNA damage, telomere-associated gene expression, and senescence-associated molecular markers. To this end, we employed oxidative stress-related experimental models, including H_2_O_2_-induced HT22 hippocampal cells and a restraint-stressed mouse model. Furthermore, we analyzed alterations in the cecal microbiome to identify microbial shifts associated with these host molecular changes, thereby providing insight into the relationship between HY7715 administration, stress-altered gut microbiota, and neurobiological aging-related responses.

## Materials and Methods

### Preparation of Probiotics

*Lactiplantibacillus plantarum* HY7715, originally isolated from kimchi, was cultured in de Man, Rogosa, and Sharpe (MRS) broth (Difco, USA) at 37°C for 20 h. After incubation, bacterial cells were harvested by centrifugation at 3,000 × g for 15 min, and the supernatant was discarded. The bacterial pellet was washed twice with phosphate-buffered saline (PBS) and resuspended in PBS. *L. plantarum* KCTC 3108, obtained from the Korean Collection for Type Cultures (KCTC, Republic of Korea), was used as a comparison strain for *in vitro* assays. For *in vitro* experiments using HT22 cells, heat-killed bacterial cells were used. Washed bacterial pellets were heat-inactivated at 90°C for 20 min and adjusted to a concentration equivalent to 10^6^ CFU/mL based on the viable count before heat inactivation. Bacterial lysates and culture supernatants were not used in this study. For the *in vivo* experiment, live HY7715 cells were prepared and orally administered at 1 × 10^9^ CFU/kg/day.

### HT22 Cell Culture

HT22 mouse hippocampal neuronal cells were obtained from the American Type Culture Collection (ATCC, USA). The cells were maintained in Dulbecco’s Modified Eagle’s Medium (DMEM) supplemented with 10% fetal bovine serum (FBS) and 1% penicillin–streptomycin (P/S) at 37°C in a humidified atmosphere of 5% CO_2_.

### Bacterial Pretreatment of HT22 Cells

For HT22 cell experiments, heat-killed HY7715 or KCTC 3108 cells were used at a concentration equivalent to 10^6^ CFU/mL based on the viable count before heat inactivation. HT22 cells were pretreated with heat-killed bacterial cells for 6 h, followed by exposure to 200 μM H_2_O_2_ for 24 h. The same bacterial pretreatment and H_2_O_2_ exposure conditions were applied for ROS, 8-OHdG, SA-β-gal staining, and qRT-PCR analyses. Cell viability under the bacterial pretreatment condition was assessed to confirm that heat-killed bacterial exposure did not cause overt cytotoxicity.

### Measurement of Reactive Oxygen Species (ROS)

Intracellular ROS levels were assessed using 2′,7′-dichlorofluorescein diacetate (DCFH-DA; Sigma-Aldrich, USA). HT22 cells were seeded in 96-well plates at a density of 5 × 10^4^ cells/well and cultured overnight. Cells were pretreated with HY7715 (10^6^ CFU/mL) for 6 h, followed by exposure to 200 μM H_2_O_2_ for 24 h. Following the bacterial pretreatment and H_2_O_2_ exposure described above, the cultures were washed twice with PBS and incubated in serum-free medium containing 10 μM DCFH-DA at 37°C for 30 min in the dark. Intracellular ROS accumulation was visualized using a ZOE^TM^ Fluorescent Cell Imager (Bio-Rad Laboratories, USA). Representative fluorescence images were acquired under identical exposure conditions across groups.

### Measurement of 8-Hydroxy-2′-Deoxyguanosine (8-OHdG)

HT22 cells were treated under the same bacterial pretreatment and H_2_O_2_ exposure conditions described in the ROS assay. To evaluate oxidative DNA damage -associated changes, the levels of 8-OHdG released into the culture medium were measured. Briefly, the HT22 cell culture supernatant was collected and centrifuged to remove any cell debris. The concentration of 8-OHdG in the supernatant was then quantified with an ELISA kit (ab285254; Abcam, UK). Absorbance was measured at 450 nm, and the concentrations were calculated from a standard curve.

### Senescence-Associated β-Galactosidase (SA-β-gal) Staining

Senescence-associated changes were assessed using an SA-β-gal staining kit (Cell Signaling Technology, USA) according to the manufacturer’s instructions. SA-β-gal–positive cells were identified by the presence of blue staining and visualized under a light microscope. The percentage of the SA-β-gal–positive area was quantified using ImageJ software (NIH, USA).

### Quantitative Real-Time PCR (qRT-PCR)

Total RNA was extracted from HT22 cells and mouse hippocampus tissues using the Easy-spin Total RNA Extraction Kit (iNtRON Biotechnology, Republic of Korea) following the manufacturer’s instructions. Complementary DNA (cDNA) was synthesized using the Omniscript Reverse Transcription Kit (Qiagen, Germany). qRT-PCR was carried out with TaqMan™ Gene Expression Assays (Applied Biosystems, USA). The following mouse-specific TaqMan probes were used: *p21* (Mm04205640_g1), *p53* (Mm01731290_g1), *Tert* (Mm00436931_m1), *Trf1* (Mm00436928_m1), *Trf2* (Mm01253555_m1), *Tnf* (Mm00443258_m1), *Il-6* (Mm00446190_m1), *Gpx1* (Mm04207457_g1), and *Sod1* (Mm01344233_g1). The gene expression was normalized using *Gapdh* (Mm99999915_g1), and relative quantification was calculated using the 2^-ΔΔCt^ method.

### Animal Study Design

**Fig. 1:**
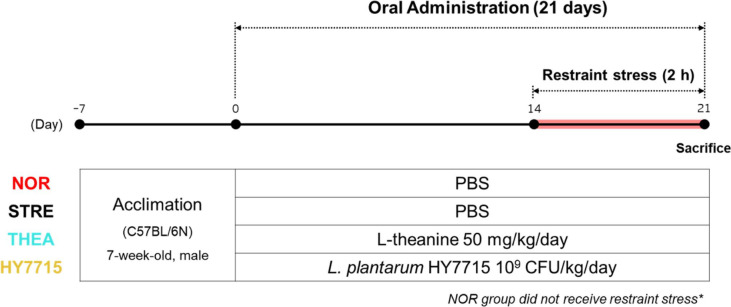
Schematic diagram of the restraint stress-induced mouse model.

The *in vivo* experiment was conducted to further validate the anti-senescent effects of HY7715 in the brain, as depicted in the experimental schema (Fig. 1). Seven-week-old male C57BL/6N mice were purchased from DBL (Republic of Korea). Mice were acclimated for 1 week under controlled environmental conditions (12-h light/dark cycle, temperature of 19-25ºC, and relative humidity of 30-70%). After acclimatization, the mice were randomly divided into four groups (n = 6 per group): NOR (non-restraint stress), STRE (restraint stress), THEA (restraint stress + L-theanine at 50 mg/kg/day), and HY7715 (restraint stress + *L. plantarum* HY7715 at 1 × 10^9^ CFU/kg/day). Treatments were orally administered for 21 days, and restraint stress was applied for 2 h/day during the final 7 days. L-theanine was used as a positive reference compound because it has been reported to attenuate restraint stress-associated behavioral, hormonal, and oxidative stress-related alterations in experimental animals [17]. All experimental procedures were conducted under IACUC approval (24E102).

### Immunohistochemistry (IHC)

To visualize the expression of p21 protein in the brain tissues, 4-μm-thick paraffin-embedded sections were deparaffinized in xylene and rehydrated through a graded series of ethanol. Antigen retrieval was performed by heating the tissue sections in 10 mM sodium citrate buffer (pH 6.0). Endogenous peroxidase activity was quenched with 3% hydrogen peroxide (H_2_O_2_), followed by blocking with 5% bovine serum albumin (BSA). Sections were then incubated overnight at 4°C with a primary antibody against p21 (1:1500, ab188224; Abcam). After washing with PBS, the sections were incubated with an HRP-conjugated secondary antibody (ab6721, Abcam) for 1 h at room temperature. Following the secondary antibody incubation, a 3,3’-diaminobenzidine (DAB) staining kit was used to visualize the immunoreactivity, and the sections were counterstained with hematoxylin. Images of the stained sections were acquired under a light microscope. For quantification, p21-positive cells in the dentate gyrus were counted and normalized to tissue area (cells/mm²).

### 16S rRNA Gene Sequencing and Microbiome Analysis

The analysis of cecal DNA samples was carried out at Macrogen (Republic of Korea). Total genomic DNA was extracted from the cecum, and the microbial community composition was analyzed by 16S rRNA gene sequencing using the MiSeq i100 platform (Illumina, USA). The V3–V4 region of the bacterial 16S rRNA gene was amplified using universal primers (V3-F; 5’-TCGTCGGCAGCGTCAGATGTGTATAAGAGA CAGCCTACGGGNGGCWGCAG-3’ and V4-R; 5’-CTCGTGGGCTCGGAGATGTGTATAAGAGACAGGACTACHVGGTATCTAATCC-3’).

Microbiome bioinformatics analysis was conducted using QIIME 2 (version 2023.9). Raw reads were quality-filtered and denoised into amplicon sequence variants (ASVs) using DADA2. For selected ASVs, species-level annotation was additionally assessed using BLASTn against the NCBI nucleotide database. Species names were assigned only when the best BLASTn hit met stringent criteria of ≥99% sequence identity and ≥98% query coverage and showed no ambiguous top match to other species. Alpha diversity (Faith’s PD) and beta diversity (weighted UniFrac distances via PCoA) were evaluated, with structural variations tested by PERMANOVA. Taxa with ≥ 1% relative abundance in any group were defined as major taxa. Differentially abundant biomarkers were identified using LEfSe (LDA score > 2.0). Spearman’s rank correlations between specific gut microbes and biochemical parameters were calculated in R (version 3.6.6). The sequencing datasets are available in the NCBI SRA repository (accession no. PRJNA1450727).

### Statistical Analysis

All data are presented as the mean ± standard deviation (SD). Statistical comparisons were performed using one-way analysis of variance (ANOVA) followed by Tukey’s multiple comparison test (version 6; GraphPad Software, USA). A *p*-value < 0.05 was considered statistically significant.

## Results

### HY7715 Attenuates Oxidative Stress, DNA Damage, and Senescence-Associated Changes in H_2_O_2_-Induced HT22 Cells

**Fig. 2:**
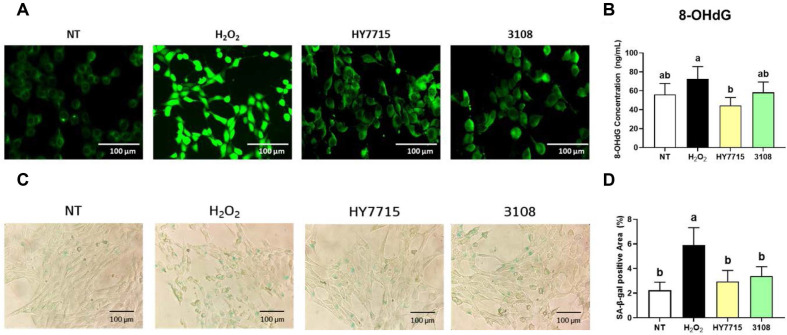
Effects of HY7715 on oxidative damage and senescence-associated changes in H_2_O_2_-induced HT22 hippocampal cells. (**A**) Intracellular reactive oxygen species (ROS) accumulation was visualized by DCFH-DA fluorescence imaging. (**B**) Oxidative DNA damage was assessed by measuring 8-hydroxy-2’-deoxyguanosine (8-OHdG) levels. (**C**) Representative images and (**D**) quantitative analysis of senescence-associated β-galactosidase (SA-β-gal) staining. Scale bar = 100 μm. Data are presented as mean ± SD. Different letters indicate significant differences (*p* < 0.05).

To evaluate the intracellular ROS levels in HT22 cells, we used the fluorescent probe DCFH2-DA. As shown in the representative images, the non-treated (NT) cells displayed minimal basal green fluorescence, indicating low levels of constitutive ROS. After exposure to H_2_O_2_, bright green fluorescence was markedly increased across the cells, suggesting enhanced intracellular ROS accumulation. On the other hand, pre-treatment with HY7715 visibly suppressed the green fluorescence signal. A similar protective effect was also observed in the cells pretreated with KCTC 3108 in this study. Collectively, these observations suggest that HY7715 attenuates intracellular ROS accumulation under oxidative stress conditions (Fig. 2A).

Since excessive ROS production directly leads to oxidative DNA damage, we subsequently measured the levels of 8-OHdG, a well-known biomarker for DNA oxidation. As expected, H_2_O_2_ treatment elevated the 8-OHdG concentration to 72.36 ng/mL compared to the NT group (56.15 ng/mL). However, pre-treatment with HY7715 significantly reduced the release of 8-OHdG to 44.54 ng/mL. In contrast, KCTC 3108 showed a smaller decrease in the 8-OHdG concentration to 58.28 ng/mL (Fig. 2B).

Given the close relationship between oxidative DNA damage and senescence-associated cellular changes, we next evaluated the effect of HY7715 on SA-β-gal activity, a commonly used marker of senescence-like responses. As depicted in Fig. 2C, H_2_O_2_ exposure markedly increased the proportion of blue-stained cells compared to the NT group, suggesting the induction of senescence-associated cellular alterations. However, pre-treatment with HY7715 visibly suppressed the accumulation of these blue-stained senescent cells. Consistent with these visual observations, quantitative analysis of the SA-β-gal positive area revealed that H_2_O_2_ treatment significantly increased the senescent area to 5.90% compared to 2.23% in the NT group. Notably, treatment with HY7715 significantly reduced this senescent area to 2.95%. KCTC 3108 also reduced SA-β-gal-positive staining (Fig. 2D).

### HY7715 Modulates Gene Expression associated with Cellular Senescence, Telomere Maintenance, and Inflammation in H_2_O_2_-Induced HT22 Cells

**Fig. 3:**
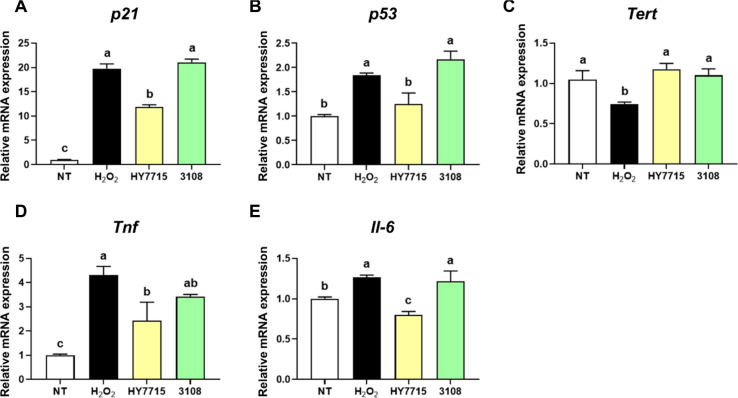
Effects of HY7715 on the expression of senescence- and inflammation-related genes in H_2_O_2_-induced HT22 cells. The mRNA expression levels of (**A**) *p21*, (**B**) *p53*, (**C**) *Tert*, (**D**) *Tnf*, and (**E**) *Il-6* were evaluated using quantitative real-time PCR (qRT-PCR). Data are presented as mean ± SD. Different letters indicate significant differences (*p* < 0.05).

Under H_2_O_2_-induced oxidative stress conditions, the mRNA expression of the senescence-associated marker *p21* was markedly increased in HT22 cells compared with the untreated control. HY7715 treatment significantly attenuated this increase, whereas the type strain KCTC 3108 did not significantly suppress *p21* expression (Fig. 3A). Similarly, the mRNA expression level of *p53* was increased to 1.84-fold following H_2_O_2_ exposure. HY7715 treatment significantly reduced *p53* expression compared to the H_2_O_2_-treated group, resulting in a 1.25-fold level relative to the NT group, whereas KCTC 3108 treatment did not result in a significant reduction (Fig. 3B).

Exposure of HT22 cells to H_2_O_2_ resulted in altered expression of telomere-associated genes. Among them, the levels of *Tert* significantly decreased to 0.75-fold compared with the untreated control. HY7715 treatment significantly attenuated this H_2_O_2_-induced decrease in *Tert* mRNA expression, increasing it to 1.17-fold. Treatment with KCTC 3108 also increased *Tert* expression to 1.10-fold compared with the H_2_O_2_-treated group (Fig. 3C). The mRNA expression of *Trf1* showed a slight decrease to 0.99-fold following H_2_O_2_ exposure; however, this change was not statistically significant. In contrast, HY7715 treatment significantly increased *Trf1* expression to 1.19-fold. Similarly, the expression level of *Trf2* was not significantly altered by H_2_O_2_ exposure, remaining at 0.98-fold compared with the untreated control. In contrast, both HY7715 and KCTC 3108 treatments significantly increased *Trf2* expression, reaching 1.42- and 1.46-fold, respectively (Fig. S1A and S1B).

Furthermore, H_2_O_2_ exposure led to increased expression of the pro-inflammatory cytokines *Tnf* and *Il-6* in HT22 cells. Notably, *Tnf* mRNA level was significantly increased to 4.31-fold compared with the untreated control. This elevation was significantly mitigated by HY7715 treatment, which reduced *Tnf* expression to 2.42-fold, whereas the type strain KCTC 3108 produced a more modest reduction to 3.42-fold. A similar pattern was observed for *Il-6* expression. Following H_2_O_2_ stimulation, *Il-6* expression increased to 1.27-fold. HY7715 treatment significantly suppressed this response, decreasing *Il-6* mRNA levels to 0.80-fold, while KCTC 3108 treatment resulted in a slight reduction to 1.22-fold (Fig. 3D and 3E).

### HY7715 Suppresses p21 Protein Expression in the Brain Tissues of Restraint-Stressed Mice

**Fig. 4:**
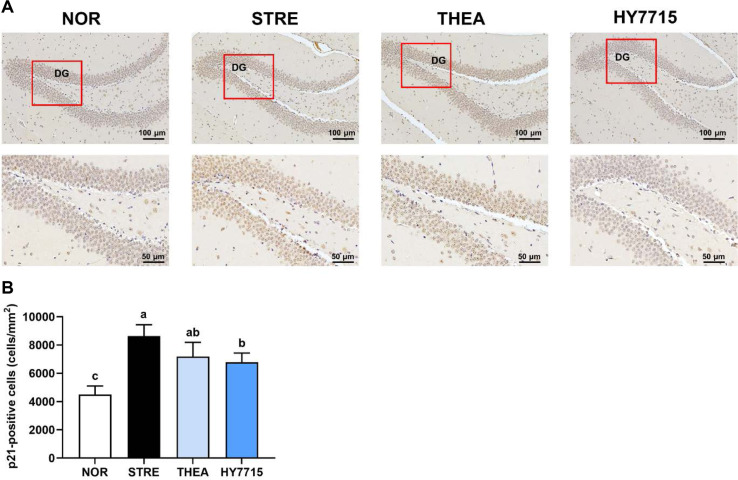
Effect of HY7715 on *p21* expression in the hippocampal tissue of restraint-stressed mice. (**A**) Representative immunohistochemical (IHC) staining images of p21-positive cells in the hippocampal dentate gyrus (DG). (**B**) Quantitative analysis of the number of p21-positive cells per unit area (cells/mm^2^). Scale bar = 100 μm (50 μm for magnified images). Data are presented as mean ± SD. Different letters indicate significant differences (*p* < 0.05).

We performed immunohistochemical (IHC) staining to visualize p21-positive cells in the dentate gyrus (DG) of the hippocampus. As shown in the representative images, STRE group showed an increased number of p21-positive cells compared with the NOR group. However, HY7715 administration attenuated this stress-induced p21 immunoreactivity, maintaining a lower number of positive cells than the STRE group (Fig. 4A).

Quantitative analysis was performed to confirm the effect of HY7715. The STRE group exhibited a significant increase in the number of p21-positive cells to 8640.42 cells/mm^2^ relative to the NOR group of 4500.75 cells/mm^2^. In contrast, the HY7715 treatment significantly reduced this number to 6787.51 cells/mm^2^. The THEA group also showed a lower mean value than the STRE group; however, this difference was not statistically significant (Fig. 4B).

### HY7715 Modulates Gene Expression Related to Cellular Senescence, Telomere Maintenance, Inflammation, and Oxidative Stress in Restraint-Stressed Mice

**Fig. 5:**
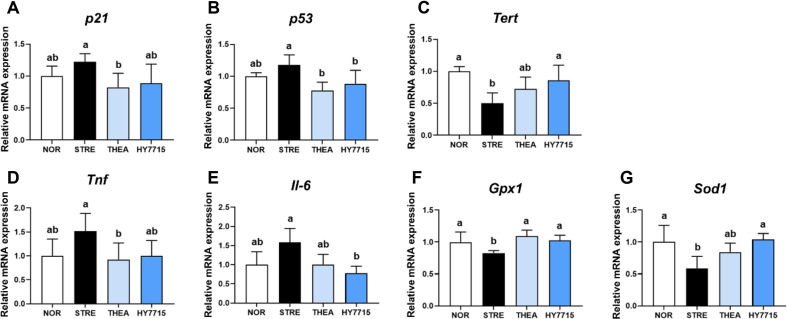
Effect of HY7715 on the expression of senescence-, inflammation-, and antioxidant-related genes in restraint-stressed mice. The mRNA expression levels of (**A**) *p21*, (**B**) *p53*, (**C**) *Tert*, (**D**) *Tnf*, (**E**) *Il-6*, (**F**) *Gpx1*, and (**G**) *Sod1* were evaluated using qRT-PCR. Data are presented as mean ± SD. Different letters indicate significant differences (*p* < 0.05).

Exposure to restraint stress elevated the mRNA expression of the cellular senescence-associated genes, *p21* and *p53*, to 1.22- and 1.18-fold, respectively, compared with the NOR group. HY7715 administration significantly attenuated this stress-induced upregulation, reducing their expression levels to 0.89- and 0.88-fold relative to the NOR group. Similarly, L-theanine treatment significantly suppressed the elevation of *p21* and *p53* compared with the STRE group (Fig. 5A and 5B).

We also evaluated the expression of telomere-associated genes, *Tert*, *Trf1*, and *Trf2*. The mRNA expression level of *Tert* was significantly reduced to 0.50-fold in the STRE group compared with the NOR group. In both the HY7715 and THEA groups, this stress-associated decrease was significantly restored to 0.86- and 0.72-fold, respectively (Fig. 5C). In contrast, while the expression of *Trf1* was modestly decreased to 0.85-fold under stress conditions, neither HY7715 nor L-theanine treatment resulted in a significant recovery. The mRNA expression of *Trf2* did not differ significantly among the experimental groups (Fig. S1C, S1D).

Regarding inflammatory cytokines, the mRNA expression levels of *Tnf* and *Il-6* were 1.51- and 1.58-fold higher, respectively, in the STRE group than in the NOR group. In the HY7715 group, *Il-6* expression was significantly reduced, whereas *Tnf* showed a decreasing trend compared with the STRE group. A similar pattern was observed in the THEA group (Fig. 5D and 5E). With respect to oxidative stress-related genes, the mRNA expression of the antioxidant genes *Gpx1* and *Sod1* was significantly reduced by restraint stress to 0.83- and 0.59-fold, respectively, compared with the NOR group. HY7715 administration significantly restored the expression of these genes to levels comparable to those of the NOR group (Fig. 5F and 5G).

### HY7715 Modulates Gut Microbiota Composition and Its Associations with Stress- and Aging-Related Molecular Parameters

**Fig. 6:**
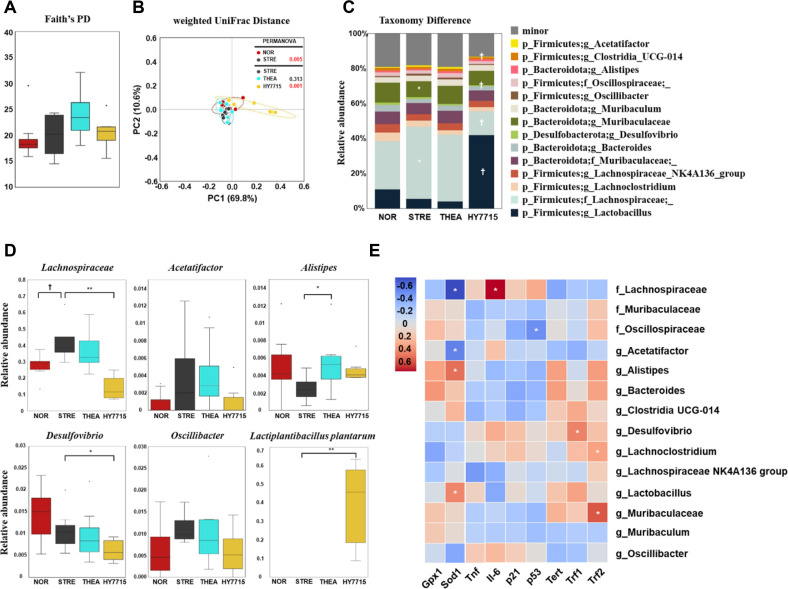
Association of HY7715 administration with gut microbiota composition in restraint-stressed mice. (**A**) Faith’s phylogenetic diversity (PD), (**B**) weighted UniFrac distances, and (**C**) taxonomic differences at the indicated levels. (**D**) Relative abundance of *Lachnospiraceae*, *Acetatifactor*, *Alistipes*, *Desulfovibrio*, *Oscillibacter*, and *Lactiplantibacillus plantarum*, and (**E**) Spearman’s correlation analysis between changes of microbiota composition and antioxidant markers, pro-inflammatory cytokines, senescence-related genes, and telomere-associated and senescence-related parameters. Asterisks indicate significant differences (*p* < 0.05).

To evaluate the impact of HY7715 administration on gut microbial composition in restraint-stressed mice, 16S rRNA gene sequencing analysis was performed. Alpha diversity, assessed using Faith’s PD index, showed no significant differences among groups, although the STRE group exhibited a slight increase compared to the NOR group (Fig. 6A). Beta diversity was evaluated using weighted UniFrac distances and visualized by PCoA. The analysis revealed significant differences in microbial community structure among groups (Fig. 6B). PERMANOVA indicated that the STRE group was significantly separated from the NOR group (*p* < 0.01), while the HY7715 administration resulted in a distinct shift in microbial composition compared to the STRE group (*p* < 0.01).

Taxonomic profiling at the family and genus levels demonstrated marked differences in microbial distribution (Fig. 6C). Restraint stress altered the microbial composition, characterized by an increased relative abundance of *Lachnospiraceae* and a decreased abundance of *Muribaculaceae*. In contrast, the relative abundance of *Lactobacillus* was markedly elevated in the HY7715-treated group. Further analysis identified specific taxa associated with stress and HY7715 intervention (Fig. 6D). At the family level, *Lachnospiraceae* was significantly increased in the STRE group compared to the NOR group (*p* < 0.05), whereas at the genus level, *Acetatifactor* and *Oscillibacter* were also elevated. Notably, the stress-associated increase in *Lachnospiraceae* was significantly reduced following HY7715 administration (*p* < 0.01). In addition, the relative abundance of *Alistipes*, which was decreased in the STRE group, was restored in the HY7715 group. Furthermore, the abundance of *Desulfovibrio* was significantly reduced in the HY7715 group compared to the STRE group (*p* < 0.05). The HY7715 group also exhibited a significant increase in ASVs assigned to *L. plantarum* compared with the STRE group.

Spearman’s correlation analysis was performed to assess the relationships between key microbial taxa and biochemical parameters related to oxidative stress, inflammation, and cellular senescence (Fig. 6E). The heatmap revealed distinct correlation patterns, with several taxa showing significant positive or negative associations (*p* < 0.05). The relative abundance of *Lactobacillus* positively correlated with the antioxidant-related gene *Sod1*. In contrast, taxa enriched under stress conditions, including *Lachnospiraceae* and *Acetatifactor*, were negatively correlated with antioxidant markers such as *Sod1*, while showing positive correlations with pro-inflammatory cytokines, particularly *Il-6*. Overall, stress-associated taxa tended to correlate positively with inflammatory and senescence-related parameters, whereas taxa enriched following HY7715 administration were associated with antioxidant-related markers.

## Discussion

Aging is a complex biological process characterized by the progressive decline of physiological integrity and represents a major risk factor for neurodegenerative diseases [18]. While diverse cellular mechanisms contribute to the progression of aging, oxidative stress caused by ROS is widely recognized as a central mediator that induces genomic instability and triggers cellular senescence [19]. In this study, we show that *L. plantarum* HY7715 attenuates oxidative stress-induced neurobiological aging-related molecular changes, accompanied by alterations in senescence-associated markers and stress-altered gut microbiota. Using both H_2_O_2_-induced HT22 hippocampal cells and a restraint-stressed mouse model, we found that HY7715 reduces oxidative damage-associated markers, modulates p53/p21-related gene expression, and partially recovered telomere-associated transcript changes.

Our *in vitro* findings provide evidence that HY7715 attenuates cellular senescence by mitigating early genomic damage induced by oxidative stress. A hallmark of SIPS is the excessive accumulation of ROS, which promotes oxidative damage and disrupts cellular homeostasis [20, 21]. In our HT22 cell model, H_2_O_2_ exposure resulted in substantial intracellular ROS generation and a marked increase in 8-OHdG levels. As a well-established biomarker of oxidative DNA damage, 8-OHdG reflects both genomic and mitochondrial DNA lesions [22]. Persistent DNA damage is known to drive cells into cell-cycle arrest, thereby promoting a senescent phenotype [23]. Pre-treatment with HY7715 significantly diminished both ROS accumulation and 8-OHdG levels, suggesting that its cytoprotective effects are associated with attenuation of oxidative damage. These results suggest that HY7715 attenuates the progression of HT22 cells toward a senescence-like phenotype by reducing oxidative damage at an early stage.

Our results suggest that the preservation of genomic integrity by HY7715 may influence downstream pathways that govern cell fate, particularly the DNA damage response (DDR). Under oxidative stress, accumulated DNA lesions activate the p53/p21 signaling cascade, thereby promoting cellular senescence [24]. This molecular response is closely linked to telomere dysfunction, as telomere shortening or structural disruption leads to the exposure of chromosomal ends, which are recognized as double-strand DNA breaks and subsequently trigger p53-dependent DDR signaling [25]. Consistent with this mechanism, HY7715 treatment was associated with reduced expression of *p53* and *p21*, suggesting a potential role in limiting the activation of senescence-related signaling pathways. These findings suggest that HY7715 may attenuate senescence-associated molecular responses not only by reducing oxidative damage but also by influencing DDR-related gene expression.

Consistent with these observations, HY7715 treatment was associated with increased expression of *Tert*, the catalytic subunit of telomerase. This was accompanied by an upregulation of *Trf2*, which plays a pivotal role in preventing the ataxia telangiectasia-mutated (ATM) kinase from recognizing the telomere end as DNA breaks [26]. Given that oxidative stress accelerates telomere attrition, the restoration of these transcripts suggests that HY7715 may influence telomere-associated regulatory pathways. These changes may be linked to attenuation of persistent DDR signaling associated with telomere dysfunction. However, as these findings are based on gene expression data, further functional validation, such as assessing telomerase enzymatic activity and quantifying absolute telomere length, will be required to fully elucidate the impact of HY7715 on telomere dynamics.

In addition, the suppression of the p53/p21 pathway by HY7715 was accompanied by a reduction in the transcriptional expression of key pro-inflammatory cytokines. Senescent cells typically reinforce the senescence state through the secretion of senescence-associated secretory phenotype (SASP) factors, including cytokines, chemokines, proteases, and growth factors [27]. Our data suggest that HY7715 reduces the mRNA expression of canonical SASP mediators, including *Tnf* and *Il-6*. Although these findings are based on intracellular transcriptional profiles rather than direct measurements of secreted proteins, they are indicative of a potential suppression of SASP-related signaling. The concurrent reduction in inflammatory markers and *p21* expression suggests that HY7715 may limit the establishment of a pro-inflammatory environment associated with cellular senescence. Taken together, these results suggest that HY7715 attenuates cellular senescence by modulating both telomere-associated pathways and SASP-related inflammatory responses, thereby potentially mitigating oxidative stress-induced neurobiological decline.

To assess whether HY7715 affects stress-induced senescence-associated changes at the tissue level, we performed immunohistochemistry (IHC) staining in the hippocampal dentate gyrus (DG), a region particularly susceptible to stress-induced functional decline [28]. HY7715 administration significantly reduced the density of p21-positive cells, which had been markedly increased by restraint stress. As p21 is a key effector of cell-cycle arrest and a well-established marker of cellular senescence [29], this reduction suggests that HY7715 attenuates the accumulation of p21-positive cells associated with stress-induced senescence-like responses in the hippocampal microenvironment. Importantly, the suppression of p21-positive cells at the tissue level is consistent with our *in vitro* findings and supports the notion that HY7715 modulates senescence-associated pathways *in vivo*. Given that senescent cells contribute to tissue dysfunction through the secretion of SASP factors, the reduction of senescent cell burden in the hippocampus may help limit local inflammatory signaling and preserve neuronal function under chronic stress conditions [30].

To validate the protective efficacy of HY7715 within a complex physiological system, we evaluated the gene expression in the brain tissues of a restraint stress-induced mouse model. Indeed, previous studies have widely demonstrated that restraint stress depletes endogenous antioxidant gene expression, triggering oxidative damage and accelerating neurobiological aging [31]. HY7715 significantly restored the antioxidant capacity, as evidenced by increased mRNA levels of *Gpx1* and *Sod1*, which were suppressed under stress conditions. These findings suggest that HY7715 may mitigate oxidative stress by enhancing endogenous antioxidant defenses, thereby limiting the activation of DNA damage-mediated aging pathways. In line with this, the expression of the senescent marker *p53* was significantly reduced, with a decreasing trend observed for *p21*. Given that oxidative stress is a key driver of *p53* activation, the restoration of antioxidant capacity is likely to attenuate stress-induced senescence signaling [32, 33]. Importantly, these results complement our *in vitro* observations, supporting a model in which HY7715 attenuates stress-induced senescence-associated molecular responses through coordinated regulation of oxidative stress- and DDR-related pathways.

Furthermore, we investigated changes in telomere-associated gene expression in the brain. HY7715 administration significantly upregulated the mRNA expression of *Tert*, which was suppressed by restraint stress. While the canonical role of *Tert* involves maintaining telomere length in proliferating cells, emerging evidence indicates that it also exerts non-canonical neuroprotective functions in post-mitotic brain tissues. Previous studies have shown that *Tert* can localize to mitochondria, where it mitigates oxidative damage and protects against neuronal apoptosis under severe stress [34, 35]. Thus, the restoration of *Tert* expression by HY7715 suggests that it may contribute to maintaining mitochondrial function and limiting oxidative stress in the brain.

Unlike our *in vitro* observations, *Trf2* expression remained stable across all *in vivo* groups. This stability is consistent with the requirement that chromosome ends must be protected from being erroneously recognized as DNA double-strand breaks, which can lead to chromosomal instability [36]. The shelterin complex, particularly through TRF2-mediated T-loop formation, plays a critical role in maintaining genomic stability by shielding telomeres from activation of DDR pathways [37]. This structural capping prevents the cellular machinery from misidentifying natural chromosome ends as sites of damage, thereby limiting inappropriate DDR activation and subsequent senescence signaling. In this context, the lack of change in *Trf2* expression suggests that major alterations in this telomere-associated factor were not evident under these conditions. Accordingly, the present data suggest that HY7715 mainly affects Tert mRNA expression rather than broadly altering shelterin-related transcripts under the current experimental conditions. However, as this study was limited to mRNA levels, further validation at the protein level and direct assessments of telomere structure will be necessary to fully elucidate these *in vivo* mechanisms.

Simultaneously, this TERT-mediated stabilization likely contributed to the mitigation of stress-induced neuroinflammation, as reflected by the reduced expression of *Il-6*. In accordance with our findings, numerous studies have highlighted that suppression of senescence-associated pathways can alleviate the SASP-related inflammatory signaling, thereby preserving neuronal function and limiting chronic neuroinflammation [38]. Collectively, these brain-specific molecular changes demonstrate that HY7715 exerts comprehensive neuroprotective effects through coordinated regulation of oxidative stress, telomere-associated pathways, and senescence-related inflammatory signaling.

Given the emerging role of the gut-brain axis in stress-related neurobiological regulation, we investigated whether the molecular effects of HY7715 were associated with alterations in the gut microbiota. Neither restraint stress nor HY7715 administration significantly affected alpha-diversity metrics, such as Faith’s PD, consistent with previous studies indicating that stress-induced dysbiosis does not necessarily reduce overall species richness [39, 40]. In contrast, beta-diversity analysis revealed significant differences in microbial community composition. Weighted UniFrac analysis suggested that restraint stress induced a marked deviation from the normal microbiota, whereas HY7715 administration further reshaped this altered community into a distinct profile. These findings indicate that HY7715 modulates microbial structure primarily through shifts in relative abundance rather than overall species richness.

Following this structural reorganization, taxonomic profiling identified specific microbial shifts associated with both restraint stress and HY7715 intervention. Among these, the most prominent change was observed in the genus *Lactobacillus*. Restraint stress markedly reduced its relative abundance, whereas HY7715 treatment significantly restored this depletion, resulting in a pronounced increase in *Lactobacillus*. This restoration may have translational relevance, as previous studies have linked reduction in *Lactobacillus* to behavioral and neurological impairments, while its recovery has been associated with functional improvements [41]. Moreover, this observation is consistent with prior reports demonstrating that HY7715 promotes the enrichment of *Lactobacillus* across different physiological models [42]. The present 16S rRNA analysis was not intended to demonstrate strain-specific HY7715 colonization, and the *L. plantarum*-related result was therefore interpreted as a taxonomic shift associated with HY7715 administration. Collectively, these findings suggest that HY7715 may contribute to reshaping the gut microbiota toward a *Lactobacillus*-enriched profile.

Correlation analysis further indicated that *Lactobacillus* abundance was positively associated with antioxidant-related markers, particularly *Sod1*, and showed an overall tendency toward inverse relationships with inflammatory and senescence-related markers. These patterns suggest a potential link between *Lactobacillus* and host oxidative stress and inflammatory regulation. Previous studies have shown that *Lactobacillus* species produce bioactive metabolites, including short-chain fatty acids (SCFAs) and neuroactive compounds such as gamma-aminobutyric acid (GABA), which may influence systemic immune responses and neuroinflammatory processes. These metabolites may influence systemic immune and oxidative stress responses, thereby potentially contributing to brain-related molecular regulation [43].

Furthermore, restraint stress induced a significant overrepresentation of the family *Lachnospiraceae*, which was attenuated following HY7715 treatment. While many members of *Lachnospiraceae* are often associated with the production of SCFAs, their functional roles are highly context-dependent, and certain taxa may opportunistically expand under severe stress conditions [44]. The positive association between stress-enriched *Lachnospiraceae* and pro-inflammatory cytokines, including *Il-6*, suggests that specific taxa within this family may contribute to peripheral inflammation under chronic stress. This highlights the functional heterogeneity within the *Lachnospiraceae* and underscores the importance of strain- or species-level resolution, which will require high-resolution metagenomic approaches to distinguish potentially pro-inflammatory pathobionts from beneficial members.

In addition to these changes, several other opportunistic taxa, including *Acetatifactor* and *Oscillibacter*, were positively associated with *Il-6*, indicating a potential link between their expansion and enhanced pro-inflammatory signaling under chronic stress conditions [45]. Given that *Il-6* is a key component of the SASP, these associations suggest that stress-responsive microbial shifts may contribute to the activation of senescence-related pathways along the gut-brain axis [46]. Similar patterns observed in *Lachnospiraceae* indicate a coordinated microbial signature associated with systemic inflammation, suggesting that multiple stress-enriched taxa may act collectively rather than independently to amplify inflammatory responses. Similarly, *Desulfovibrio* abundance was reduced under restraint stress and further decreased following HY7715 administration. However, correlation analysis revealed positive association with both inflammatory markers, including *Il-6*, and genomic stability-related markers (*Tert*, *Trf1*, and *Trf2*), indicating a complex and context-dependent relationship with host physiology. Although *Desulfovibrio* is widely recognized as a sulfate-reducing genus capable of producing hydrogen sulfide (H_2_S), its functional effects appear to be highly dependent on the host and environmental context, with both protective and detrimental roles reported [47]. In this regard, the discrepancy between abundance changes and correlation patterns suggests that *Desulfovibrio* may reflect broader host-microbiota remodeling under stress conditions rather than serving as a unidirectional pro-inflammatory taxon. In contrast, *Alistipes*, which was restored following HY7715 administration, showed positive correlations with antioxidant and genomic stability markers, including *Sod1* and *Tert*, while exhibiting a negative association with *Il-6*.

Taken together, these findings suggest that HY7715 administration is associated with a shift in gut microbiota composition from a stress-associated dysbiotic profile toward a composition linked to enhanced antioxidant capacity and reduced inflammation. Notably, the present study extends beyond descriptive microbiota alterations by linking specific microbial signatures to host senescence-related, inflammatory, and antioxidant markers, highlighting a potential association between gut microbial signatures and stress-induced neurobiological aging-related molecular changes. However, some limitations of the present study should be acknowledged. First, the restraint stress model used in this study does not fully recapitulate chronological brain aging, but rather reflects stress-induced neurobiological aging-related molecular alterations. Second, although SA-β-gal staining, p53/p21-related gene expression, inflammatory markers, and hippocampal p21 immunoreactivity collectively support senescence-associated responses, these markers do not establish irreversible cellular senescence. Third, the telomere-related findings are based on transcript-level changes, and direct measurements of telomere length, telomerase activity, or TERT/TRF protein expression were not performed. Finally, the microbiome results remain associative, and further studies integrating metagenomic, metabolomic, or functional approaches will be required to determine whether gut microbiota changes directly contribute to HY7715-associated effects.

**Fig. 7:**
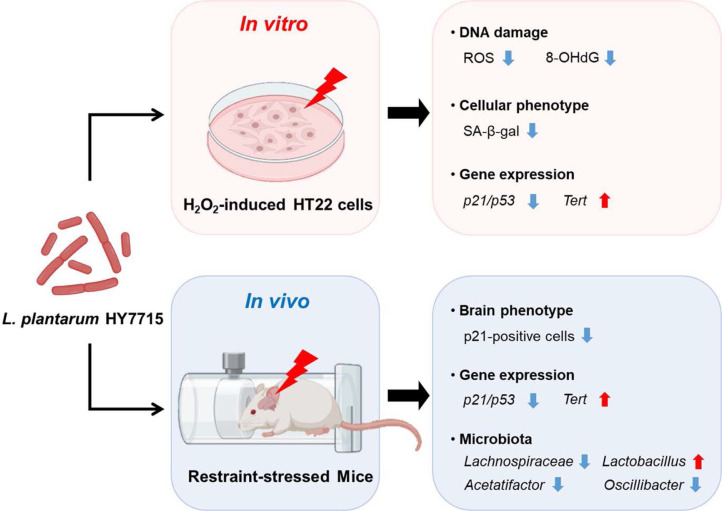
Proposed model for the effects of *Lactiplantibacillus plantarum* HY7715 on oxidative stress-induced senescence-associated molecular changes *in vitro* and *in vivo*.

In conclusion, this study suggests that *L. plantarum* HY7715 attenuates oxidative stress-induced neurobiological aging-related molecular changes, accompanied by alterations in senescence-associated markers and stress-altered gut microbiota (Fig. 7). Our findings suggest that HY7715 treatment partially recovers telomere-associated transcript changes while reducing p53/p21-related gene expression. These effects are supported by reduced oxidative damage-associated markers and partial normalization of stress-disrupted gut microbial composition.

## Supplemental Materials

Supplementary data for this paper are available on-line only at http://jmb.or.kr.
