# Supplementary material for: Lactiplantibacillus plantarum HY7715 Attenuates Oxidative Stress-Induced Neurobiological Aging-Related Changes by Modulating Senescence-Associated Markers and Gut Microbiota
Source: J Microbiol Biotechnol. 2026 Jun 2;36:e2604063. doi: 10.4014/jmb.2604.04063 (PMC13275255; doi:10.4014/jmb.2604.04063)
Supplement: Supplementary file 1 [file jmb-36-e2604063-supple.pdf]

J. Microbiol. Biotechnol. 2026. 36: e2604063
https://doi.org/10.4014/jmb.2604.04063

Supplementary Figures

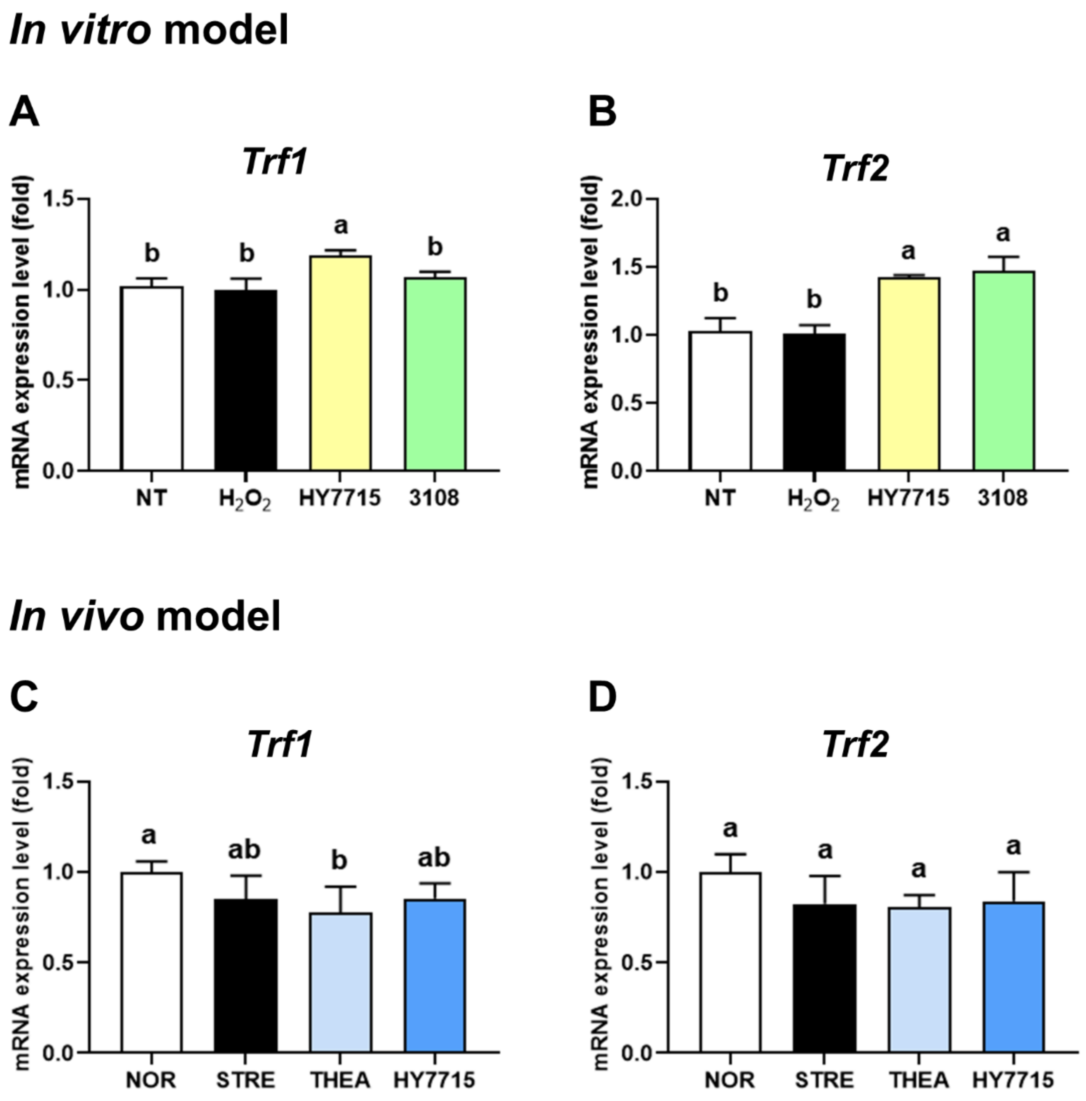

Fig. S1. Effects of HY7715 on *Trf1* and *Trf2* mRNA expression in *in vitro* and *in vivo* models. Relative mRNA expression levels of (A) *Trf1* and (B) *Trf2* in H_2O_2-induced cells treated with HY7715 or type strain (3108). Relative mRNA expression levels of (C) *Trf1* and (D) *Trf2* in brain tissues of restraint-stress mice. Data are presented mean ± SD. Different letters indicate significant differences ($p < 0.05$).

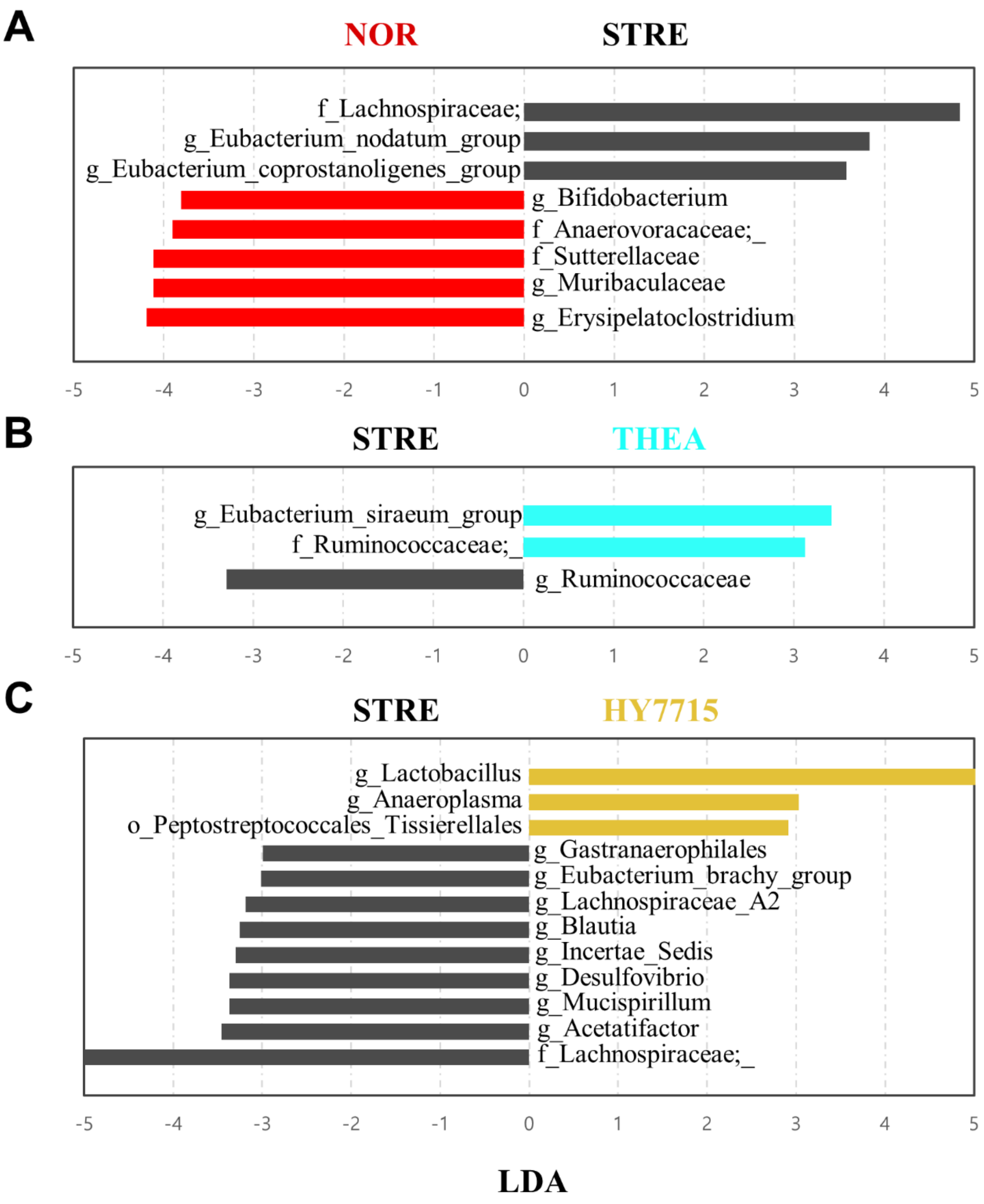

Fig. S2. **Taxonomic cladogram of differentially abundant microbial taxa identified by LEfSe analysis (LDA > 2.0 at the genus level) comparing the STRE group with (A) NOR, (B) THEA, and (C) HY7715 groups.**
